# Supplementary material for: Climate solution or corporate co-optation? US and Canadian publics’ views on agricultural gene editing
Source: PLoS One. 2022 Mar 21;17(3):e0265635. doi: 10.1371/journal.pone.0265635 (PMC8936474; doi:10.1371/journal.pone.0265635)
Supplement: S1 File — (DOCX) [file pone.0265635.s001.docx]

Perceptions of gene editing in agriculture

Start of Block: Consent

You are invited to participate in a research project exploring attitudes and perspectives on gene editing technologies in the context of agriculture. Gathering information on public views will be important to addressing the ethical and social challenges raised by these new technologies.

**Study procedures:** Your participation is entirely voluntary. If you choose to participate, you will be asked to answer a series of questions that will last about 15-20 minutes. There are no right or wrong answers, we seek your opinions only. You may terminate your participation in the study at any time.

**Sponsor:** Funding for this study is provided by a grant from Genome British Columbia.

**Potential Risks:** No psychological, cultural, privacy or confidentiality risks are anticipated or intended through this study. We do not think there is anything in this study that is harmful.

**Potential Benefits:** You may benefit from this study by having an opportunity to reflect on your views on new gene editing technologies in agriculture. Additionally, others may benefit in the future through what is learned in this study about public perspectives on gene editing technologies.

**Confidentiality:** Information that discloses your identity will not be released without your consent. All documents will be identified by a code available only to the survey company, and your identity will not be shared with the research investigators.

**Who can you contact for more information about the study?** The Principal Investigator, Dr. Terre Satterfield, is available to answer any questions you may have about this research study. You can also contact Sara Nawaz, the PhD Candidate involved with the study.

Dr. Terre Satterfield, Professor, Institute for Resources, Environment and Sustainability, Phone: 604-822-2333, email: terre.satterfield@ires.ubc.ca

Sara Nawaz, PhD Candidate, Institute for Resources and the Environment, Phone: 778-320-3985, email: sara.nawaz@ires.ubc.ca 

**Who can you contact if you have complaints or concerns about the study?** If you have any concerns or complaints about your rights as a research participant and/or your experiences while participating in this study, contact the Research Participant Complaint Line in the UBC Office of Research Ethics at 604-822-8598 or if long distance e-mail RSIL@ors.ubc.ca or call toll free 1-877-822-8598.

**Participant consent:** If the questionnaire is completed, it will be assumed that consent has been given. Your participation in this study is entirely voluntary and you may refuse to participate or withdraw from the study at any time without penalty or offense. By giving consent you do not waive any legal rights.

End of Block: Consent

Start of Block: Initial familiarity/similarity questions

Q1 Thank you again for agreeing to participate in this survey. We would like to begin by asking you some questions about your understanding of a few terms.     Please indicate whether you disagree or agree with the following statements:

|  | Strongly disagree (1) | Disagree (2) | Neutral (3) | Agree (4) | Strongly agree (5) | Don't know/ not sure (6) |
| --- | --- | --- | --- | --- | --- | --- |
| I am familiar with the term "genetically modified" or GM (1) |  |  |  |  |  |  |
| I am familiar with the term "gene editing" (2) |  |  |  |  |  |  |
| I am familiar with the term "gene drive" (3) |  |  |  |  |  |  |

| Page Break |  |
| --- | --- |

Q2 Before we go further, we will first define a few terms.   **Genetic modification** is the process of altering an organism’s genome (its full set of genes) by inserting genes from one organism into another.  **Gene editing** is the process of using a tool that works like a pair of scissors to make edits in an organism’s genes—unlike genetic modification, these edits are targeted to a specific location in the organism’s genome. **Gene drives** are a technique that ensures that an edit is inherited by the next generation and eventually spreads through that organism’s population.

 Based on these definitions, would you say that these three processes—genetic modification, gene editing, and gene drives—seem **similar to each other**, or**different from each other**?

- Very similar (1)
- Somewhat similar (2)
- Neither similar nor different (3)
- Somewhat different (4)
- Very different (5)
- Don't know/not sure (6)

Q3 Please feel free to explain your reasoning:

________________________________________________________________

________________________________________________________________

________________________________________________________________

________________________________________________________________

________________________________________________________________

End of Block: Initial familiarity/similarity questions

Start of Block: Intro to vignettes

Next, we will present you with **several examples** of ways that these technologies have been applied to agriculture.

End of Block: Intro to vignettes

Start of Block: Tomato case

Common supermarket tomatoes have been bred from older, “heirloom” varieties to be more easily picked and packaged; this has been achieved by **removing the lumpy “shoulders”** characteristic of heirloom varieties. However, **genes for sweetness and flavor were lost** during this breeding process. This change can be reversed by editing with the ‘scissors’ mentioned above: **genes from the heirloom tomato** could be **inserted into a targeted location** in the genome of the common supermarket tomato.
 (Heirloom tomatoes above; Common supermarket tomato variety below)

Q4 Please tell us your “gut feeling” or comfort level with regard to this change to tomatoes:

- Very comfortable (1)
- Comfortable (2)
- Neither comfortable nor uncomfortable (3)
- Uncomfortable (4)
- Very uncomfortable (5)
- Don't know/not sure (6)

Q5 Please feel free to explain your reasoning:

________________________________________________________________

________________________________________________________________

________________________________________________________________

________________________________________________________________

________________________________________________________________

| Page Break |  |
| --- | --- |

Q6 Now, what if it were a sweetness gene **from a sugar beet**, not an heirloom tomato that was used. 
 
How do you feel about this altered version of the application?

- Very comfortable (1)
- Comfortable (2)
- Neither comfortable nor uncomfortable (3)
- Uncomfortable (4)
- Very uncomfortable (5)
- Don't know/ not sure (6)

Q7 Please feel free to explain your reasoning:

________________________________________________________________

________________________________________________________________

________________________________________________________________

________________________________________________________________

________________________________________________________________

End of Block: Tomato case

Start of Block: Cattle case

While beef cattle do not grow horns, most dairy cattle do. As these horns can injure other cows as well as the farmers or workers that handle dairy cattle, the **horns are usually surgically removed** early in life, causing pain and suffering to the cattle. To remedy this, researchers have **isolated the “hornless” gene from beef cattle and inserted it** into the genomes of dairy cattle. The genome of dairy cattle is also altered so **this hornless gene will spread through a population**. Such hornless cattle could be bred via traditional techniques, but due to the pace of traditional breeding, it **would take much longer** to spread this gene through the dairy cattle population.

Q8 Please tell us your “gut feeling” or comfort level with regard to this change to dairy cattle:

- Very comfortable (1)
- Comfortable (2)
- Neither comfortable nor uncomfortable (3)
- Uncomfortable (4)
- Very uncomfortable (5)
- Don't know/not sure (6)

Q9 Please feel free to explain your reasoning:

________________________________________________________________

________________________________________________________________

________________________________________________________________

________________________________________________________________

________________________________________________________________

| Page Break |  |
| --- | --- |

Q10 Now, what if this approach were used to **increase the milk-producing capacity** of the next generation of cattle.    How do you feel about this altered version of the application?

- Very comfortable (1)
- Comfortable (2)
- Neither comfortable nor uncomfortable (3)
- Uncomfortable (4)
- Very uncomfortable (5)
- Don't know/not sure (6)

Q11 Please feel free to explain your reasoning.

________________________________________________________________

________________________________________________________________

________________________________________________________________

________________________________________________________________

________________________________________________________________

End of Block: Cattle case

Start of Block: Wheat case

Wheat is one of the most widely cultivated crops in the world, but it has become compromised by climate change. To address this, researchers have **edited a wheat plant** to be more **resilient to higher temperatures and prolonged droughts**. To achieve this, they have **inserted a gene from another plant species into a targeted location** in the genome of the wheat plant.

Q12 Please tell us your "gut feeling" or comfort level with regard to this change to wheat:

- Very comfortable (1)
- Comfortable (2)
- Neither comfortable nor uncomfortable (3)
- Uncomfortable (4)
- Very uncomfortable (5)
- Don't know/not sure (6)

Q13 Please feel free to explain your reasoning.

________________________________________________________________

________________________________________________________________

________________________________________________________________

________________________________________________________________

________________________________________________________________

| Page Break |  |
| --- | --- |

Q14 Now, what if instead of a staple crop like wheat, this technique was **applied to a less important crop like blueberries**.
 
 How do you feel about this altered version of the application?

- Very comfortable (1)
- Comfortable (2)
- Neither comfortable nor uncomfortable (3)
- Uncomfortable (4)
- Very uncomfortable (5)
- Don't know/not sure (6)

Q15 Please feel free to explain your reasoning:

________________________________________________________________

________________________________________________________________

________________________________________________________________

________________________________________________________________

________________________________________________________________

End of Block: Wheat case

Start of Block: Tradeoff group A

Q16A Next, we’d like to ask you a couple questions about some tradeoffs involving gene-edited crops.   First, some researchers think that growing gene-edited crops could be used to significantly reduce the volume of pesticides used on farms. This is because those crops would be edited to be resistant to specific pests, and so farms could avoid general spraying.   Assuming scientists are right about this, which of the following choices between production of gene-edited crops and pesticide use do you prefer?

- A **large increase** in production of gene-edited crops in exchange for a **large decrease** in pesticide use   (1)
- A **small increase** in production of gene-edited crops in exchange for a **small decrease** in pesticide use   (2)
- **No change:** production of gene-edited crops and pesticide use stay the same   (3)
- A **small decrease** in production of gene-edited crops, in exchange for a **small increase** in pesticide use   (4)
- A **large decrease** in production of gene-edited crops, in exchange for a **large increase** in pesticide use   (5)
- I prefer not to answer this question because I don't have information on **who owns or controls** these technologies (7)
- I prefer not to answer this question because it doesn't discuss **other ways of avoiding pesticide use** (8)
- Don't know/not sure (6)

Q17A Please feel free to add any comments that you had on this question about tradeoffs between gene-edited crops and pesticide use:

________________________________________________________________

________________________________________________________________

________________________________________________________________

________________________________________________________________

________________________________________________________________

| Page Break |  |
| --- | --- |

Q18A Some researchers also think that use of gene-edited crops could benefit the conservation of biodiversity. This is because edited versions might have higher crop yields per area of land than non-edited ones. Therefore, less land (e.g., forest habitat) would be converted to agriculture.   Assuming scientists are right about this, which of the following choices between gene-edited crop production and biodiversity do you prefer?

- A **large increase** in production of gene-edited crops in exchange for **large improvements** to biodiversity   (1)
- A **small increase** in production of gene-edited crops in exchange for **small improvements** to biodiversity   (2)
- **No change**: production of gene-edited crops and biodiversity loss stay the same   (3)
- A **small decrease** in use of gene-edited crops, in exchange for a **small loss** of biodiversity   (4)
- A **large decrease** in use of gene-edited crops, in exchange for a **large loss** of biodiversity   (5)
- I prefer not to answer this question because I don't have information on **who owns or controls** these technologies (7)
- I prefer not to answer this question because it doesn't discuss **other ways of conserving biodiversity** (8)
- Don't know/not sure (6)

Q19A Please feel free to add any comments that you had on this question about tradeoffs between gene-edited crops and biodiversity:

________________________________________________________________

________________________________________________________________

________________________________________________________________

________________________________________________________________

________________________________________________________________

End of Block: Tradeoff group A

Start of Block: Tradeoff group B

Q16B Next, we’d like to ask you a couple questions about some tradeoffs involving gene-edited crops.   First, some researchers think that growing gene-edited crops could be used to significantly reduce the volume of pesticides used on farms. This is because those crops would be edited to be resistant to specific pests, and so farms could avoid general spraying.   Assuming scientists are right about this, which of the following choices between production of gene-edited crops and pesticide use do you prefer?

- A **large decrease** in production of gene-edited crops, in exchange for a **large increase** in pesticide use   (5)
- A **small decrease** in production of gene-edited crops, in exchange for a **small increase** in pesticide use   (4)
- **No change:** production of gene-edited crops and pesticide use stay the same   (3)
- A **small increase** in production of gene-edited crops in exchange for a **small decrease** in pesticide use   (2)
- A **large increase** in production of gene-edited crops in exchange for a **large decrease** in pesticide use   (1)
- I prefer not to answer this question because I don't have information on **who owns or controls** these technologies (7)
- I prefer not to answer this question because it doesn't discuss **other ways of avoiding pesticide use** (8)
- Don't know/not sure (6)

Q17B Please feel free to add any comments that you had on this question about tradeoffs between gene-edited crops and pesticide use:

________________________________________________________________

________________________________________________________________

________________________________________________________________

________________________________________________________________

________________________________________________________________

| Page Break |  |
| --- | --- |

Q18B Some researchers also think that use of gene-edited crops could benefit the conservation of biodiversity. This is because edited versions might have higher crop yields per area of land than non-edited ones. Therefore, less land (e.g., forest habitat) would be converted to agriculture.   Assuming scientists are right about this, which of the following choices between gene-edited crop production and biodiversity do you prefer?

- A **large decrease** in use of gene-edited crops, in exchange for a **large loss** of biodiversity   (5)
- A **small decrease** in use of gene-edited crops, in exchange for a **small loss** of biodiversity   (4)
- **No change**: production of gene-edited crops and biodiversity loss stay the same   (3)
- A **small increase** in production of gene-edited crops in exchange for **small improvements** to biodiversity   (2)
- A **large increase** in production of gene-edited crops in exchange for **large improvements** to biodiversity   (1)
- I prefer not to answer this question because I don't have information on **who owns or controls** these technologies (7)
- I prefer not to answer this question because it doesn't discuss **other ways of conserving biodiversity** (8)
- Don't know/not sure (6)

Q19B Please feel free to add any comments that you had on this question about tradeoffs between gene-edited crops and biodiversity:

________________________________________________________________

________________________________________________________________

________________________________________________________________

________________________________________________________________

________________________________________________________________

End of Block: Tradeoff group B

Start of Block: Independent variable scales

| 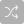 |
| --- |

Q20 Next, we will ask you about your views on related topics. Please indicate whether you disagree or agree with the following statements about trust:

|  | Strongly disagree (1) | Disagree (2) | Neutral (3) | Agree (4) | Strongly agree (5) | Don't know/not sure (6) |
| --- | --- | --- | --- | --- | --- | --- |
| I trust scientists to adequately manage the risks associated with genetic technologies (1) |  |  |  |  |  |  |
| I trust regulators to make sure the risks of genetic technologies are minimized (2) |  |  |  |  |  |  |
| I trust agricultural companies to be conscious of their responsibilities in using genetic technologies (3) |  |  |  |  |  |  |
| I trust environmental activists to inform me about any risks of genetic technologies (4) |  |  |  |  |  |  |

| Page Break |  |
| --- | --- |

| 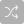 |
| --- |

Q22 Please indicate whether you disagree or agree with the following statements about technologies in general:

|  | Strongly disagree (1) | Disagree (2) | Neutral (3) | Agree (4) | Strongly agree (5) | Don't know/not sure (6) |
| --- | --- | --- | --- | --- | --- | --- |
| Hope for the future lies more in people than in technology (1) |  |  |  |  |  |  |
| The bad effects of technology outweigh its advantages (2) |  |  |  |  |  |  |
| Future resource shortages will be solved by technology (3) |  |  |  |  |  |  |
| A country’s progress can be measured by its technological development (4) |  |  |  |  |  |  |
| We are worse off than ever, because of the burden of new technologies (5) |  |  |  |  |  |  |

| Page Break |  |
| --- | --- |

| 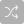 |
| --- |

Q21 Now, please indicate where you disagree or agree with the following statements about climate change:

|  | Strongly disagree (1) | Disagree (2) | Neutral (3) | Agree (4) | Strongly agree (5) | Don't know/ not sure (6) |
| --- | --- | --- | --- | --- | --- | --- |
| Climate change poses new risks to forest ecosystems (e.g., extreme natural hazards such as fire, temperature or drought) (1) |  |  |  |  |  |  |
| Scientists agree that the evidence for human-caused climate change is partial at best (2) |  |  |  |  |  |  |
| The unique problems of climate change necessitate more caution than action (3) |  |  |  |  |  |  |
| Human impacts are causing changes at a planetary scale that are destabilizing the functioning of the Earth’s systems (4) |  |  |  |  |  |  |
| A future scientist hundreds of years from now will see this period as the great climate extinction event (5) |  |  |  |  |  |  |
| Climate change will affect some ecosystems so quickly and strongly that large-scale ecosystem changes will follow (6) |  |  |  |  |  |  |
| Many other problems that also impact people globally are more urgent than climate change (7) |  |  |  |  |  |  |

| Page Break |  |
| --- | --- |

| 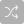 |
| --- |

Q23 These next questions involve opinions about substances or contaminants in everyday life. Please indicate whether you disagree or agree with the following statements:

|  | Strongly disagree (1) | Disagree (2) | Neutral (3) | Agree (4) | Strongly agree (5) | Don't know/not sure (6) |
| --- | --- | --- | --- | --- | --- | --- |
| Naturally occurring hazardous substances are just as worrisome to me as human-made ones (1) |  |  |  |  |  |  |
| If soil already has a natural level of a substance like nitrogen in it, it probably won’t matter if the concentrations of that substance increase (2) |  |  |  |  |  |  |
| Most people are exposed to so many pollutants every day and remain healthy, so constant low levels of exposure are probably safe (3) |  |  |  |  |  |  |
| Human bodies are slowly evolving to be less tolerant of environmental pollution (4) |  |  |  |  |  |  |
| It is possible to gain immunity to chemicals, if exposed to low levels for a long time (5) |  |  |  |  |  |  |
| Human bodies are fragile, and so have a poor capacity to withstand toxins (6) |  |  |  |  |  |  |

| Page Break |  |
| --- | --- |

| 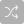 |
| --- |

Q24 Next we’ll ask you some questions about your thoughts on globalization and corporations. Please indicate whether you disagree or agree with the following statements:

|  | Strongly disagree (1) | Disagree (2) | Neutral (3) | Agree (4) | Strongly agree (5) | Don't know/not sure (6) |
| --- | --- | --- | --- | --- | --- | --- |
| The increasing influence of large corporations is a problem (1) |  |  |  |  |  |  |
| Globalization has positive impacts for the large majority of people (2) |  |  |  |  |  |  |
| I understand that corporations try to make money, but I don’t think they should control knowledge through patents (3) |  |  |  |  |  |  |
| Global tech monopolies such as Amazon, Apple and Facebook should be broken up (4) |  |  |  |  |  |  |

| Page Break |  |
| --- | --- |

| 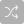 |
| --- |

Q25 Next, we’ll ask you a few questions about the Green Revolution (~1950-1970), which included a large increase in crop production in developing countries (e.g., India). This increase was because of modern agricultural techniques including things such as fertilizers and pesticides. Please indicate whether you disagree or agree with the following statements:

|  | Strongly disagree (1) | Disagree (2) | Neutral (3) | Agree (4) | Strongly agree (5) | Don't know/not sure (6) |
| --- | --- | --- | --- | --- | --- | --- |
| I was familiar with the meaning of the term “the Green Revolution” before reading this question (1) |  |  |  |  |  |  |
| The Green Revolution was a positive development for farmers in countries like India (2) |  |  |  |  |  |  |
| Because of the Green Revolution, many fewer people starved or suffered hunger than otherwise would have (3) |  |  |  |  |  |  |
| The Green Revolution led to big losses of traditional crops & agricultural biodiversity (4) |  |  |  |  |  |  |
| The Green Revolution brought much-needed increases in agricultural productivity (5) |  |  |  |  |  |  |
| The Green Revolution has exacerbated inequalities amongst farmers (6) |  |  |  |  |  |  |
| The Green Revolution was not necessary; such advances in productivity could have occurred in a more environmentally sustainable manner (7) |  |  |  |  |  |  |
| The Green Revolution has contributed to the excessive use of pesticides and fertilizers in modern farming (8) |  |  |  |  |  |  |

End of Block: Independent variable scales

Start of Block: Independent variables--demographics

Q26 Great! We’re almost done. To close, we’d like to ask you a final few questions about yourself.   

 What is your gender?

- Female (1)
- Male (2)
- Nonbinary (3)
- Trans or transitioning (4)
- If these categories do not accurately reflect how you self-identify, use this space to write your response: (5) ________________________________________________

Q27 What is your birth year?

▼ 1920 (1) ... 2002 (83)

Q28 What is the highest level of formal education you have completed?

- Doctorate (PhD or EdD) or Professional (MD, JD, DVM, DDS) (1)
- Masters (MA, MS, MBA, Med) (2)
- Bachelors (BA, BS, AB) (3)
- Associates (2-year degree) (4)
- Some college, no degree (5)
- High school diploma or GED (6)
- 9th – 12th grade, no diploma (7)
- 8th grade or less (8)

Q29 Where would you place yourself on the following political spectrum?

- Very liberal (1)
- Liberal (2)
- Moderate (3)
- Conservative (4)
- Very conservative (5)

Q30 What is your race or ethnic background? Select all that apply:

- American Indian, First Nation, or Pacific Islander (3)
- Black (2)
- East Asian (1)
- Hispanic (4)
- Middle Eastern (9)
- South Asian (8)
- White (6)
- If these categories do not accurately reflect how you self-identify, use this box to write your response (7) ________________________________________________

| Page Break |  |
| --- | --- |

Q31 Almost there, just two last questions. Are you currently working for pay or something else?

- Working for pay (1)
- On vacation or sick leave from regular job (2)
- Unpaid household work (children, family, etc.) (3)
- Going to school (4)
- Looking for work (5)
- Unable to work, differently abled (6)
- Retired (7)
- Other (add details here) (8) ________________________________________________

Q32 How often do you attend religious services?

- More than once a week (1)
- Once a week (2)
- Once or twice a month (3)
- Rarely (4)
- Never (5)

Q33 What is your yearly income?

- Less than $20,000 (1)
- $20,000-$30,000 (2)
- $30,000-$40,000 (3)
- $40,000-$50,000 (4)
- $60,000-$80,000 (5)
- $80,000-$100,000 (6)
- Greater than $100,000 (7)
- I prefer not to say (9)

End of Block: Independent variables--demographics

Start of Block: End of survey message

You have reached the end of the survey. Thank you very much for participating—we really appreciate your input.

If you have questions or concerns, please feel free to contact us:

Terre Satterfield: [terre.satterfield@ires.ubc.ca](mailto:terre.satterfield@ires.ubc.ca)

Sara Nawaz: sara.nawaz@ires.ubc.ca

If you have any complaints you may contact UBC’s Office of Research at RSIL@ors.ubc.ca.

End of Block: End of survey message
