# Supplementary material for: Climate solution or corporate co-optation? US and Canadian publics’ views on agricultural gene editing
Source: PLoS One. 2022 Mar 21;17(3):e0265635. doi: 10.1371/journal.pone.0265635 (PMC8936474; doi:10.1371/journal.pone.0265635)
Supplement: Attachment — Submitted filename: Response to reviewers_Plos one January 19 2022.docx [file pone.0265635.s002.docx]

Response to reviewers

Dear editors and reviewers,

Thank you for your interest in our manuscript, and for your helpful feedback (particularly Reviewer 2). The revised manuscript includes the following changes:

- We have significantly reworked the introductory sections to make the case for the need to understand which views (proponents’ vs. critics’) most resonate with public groups; to better situate our work in the risk perceptions literature (specifically, we incorporate critiques of gene editing from qualitative and small-groups work that have heretofore been excluded from risk perceptions literature); and to better highlight our novel contribution of studying gene editing views in relation to the urgency of climate change and critiques of industrial food systems.
- We sought to clarify the term we originally used, ‘system critical’, abandoning this cryptic wording for, simply: views that are critical of industrial food systems.
- We have also shifted away from dichotomous ‘accept or reject’ framings to emphasize that the goal of our manuscript is to explore different factors influencing overall views on gene editing.
- We have incorporated several helpful reference suggestions from Reviewer 2, which also support the reworking of the introductory framing of our paper (as well as the policy discussion).
- We have clarified the term ‘proponents’ and worked to be more specific in its use.
- We have edited and/or expanded our discussion and policy implications section so as to better connect our findings with the broader perceptions literature, and as well, to suggest how that field of research might better reflect principles of responsible innovation.

These, and other more minor points, are discussed below in more detail.

Thank you for your consideration.

Sara Nawaz

Terre Satterfield

| Reviewer | Comment | Response |
| --- | --- | --- |
| 1 | Much of this research is based on that the potential for gene editing to be viewed in a slightly more optimistic light – was there any information provided or questions in the survey to ensure the surveyor understands the difference between GM and GE? | We describe in Section 2.1 that we had included a question at the outset of the survey assessing participants’ familiarity with various concepts of genetic engineering. We also explain that we included a question asking participants whether these items, including GM and gene editing, appeared similar or different to them, and gave them a free response section to note any comments on this question. We did find more familiarity, unsurprisingly, with GM vs. gene editing, but we also found that the differences appeared generally well understood once we provided definitions (as judged based on the free response reflections provided). |
| 2 | The title needs to be changed. It does not well reflect the content of the paper. | We have changed the title to: “Climate solution or corporate co-optation? US and Canadian publics’ views on agricultural gene editing”, which we feel reflects the revisions we have undertaken here, particularly our efforts to reframe the paper as incorporating broader societal dilemmas and challenges into perceptions work on gene editing. |
|  | The term ‘system critical’ is used throughout but it’s not explained and inconsistently kept in ‘’. I suggest this term is removed from the paper and instead the authors find another way to describe what they mean by this phrase. Even by the end of the paper, I don’t feel I know what the authors are referring to with it. | We have removed this term from the manuscript, and instead better explain that we refer to (political economic) critiques of industrial food systems. We abbreviate this in various ways throughout the manuscript, such as “critiques of food systems”, but hope that this initial definition makes these references easier to follow. We also now explain the background for this concept in more depth and with more clarity, highlighting these critiques specifically in the qualitative literature on genetic engineering. We also foreground the term more fully in the introductory discussion of this paper’s contribution. |
|  | The front end of the paper needs to be rewritten to frame the paper, provide more accuracy about the current empirical realities, and situate the paper in an academic theoretical literature. The authors need to make clear what their contribution is and to which debate. Is this a paper that contributes to public perceptions literature or the responsible innovation literature? As it is, the paper seems to rush into the empirical work without taking to time and space to let the reader know why it matters theoretically and for policy. Why does this paper matter? What's the point you're trying to make? | We have significantly reworked the introductory section of the manuscript to address this important point. As we see it, our introductory section now frames the manuscript in the context of opposing understandings of gene editing put forward by proponents and critics, respectively. As publics grapple with the novelty of a new suite of technologies, it is unclear—which perspective resonate, climate benefits or food system critiques (and are the two mutually exclusive)?  Furthermore, we clarify that our primary theoretical contribution is to introduce a novel variable that might predict views on gene editing (or genetic engineering more broadly, perhaps)—that of views on industrial food systems. As we explain in the manuscript, much qualitative work has highlighted the importance of more structural or system-level questions, such as whether a technology operates within a monocultural system vs. agroecological one. As far as we are aware, this is the first effort to bring such important considerations into a quantitative risk perceptions study.  We also note that we reworked the order in which we discuss hypotheses/propositions, and feel that the new order better supports the flow of the theoretical framing and context. |
|  | The first paragraph needs more precision and accuracy. Who are the proponents? This sentence needs evidencing as it’s a crucial motivation for the paper – ‘Proponents also argue that the precision of edits and ability to produce non-transgenics render edited organisms less risky and less controversial than genetically modified organisms (GMOs), and thus more appealing to consumers’. Where the authors talk about proponents, I would encourage them to either name them or talk about the types of actors they are throughout the paper. | Thank you for this useful suggestion. We have ensured that ‘proponents’ is clarified in its first instance as including technology developers, agricultural companies, and policymakers, amongst others. We have modified our use of proponents where possible throughout the paper to better specify who we are referring to (e.g., “proponents, such as academic researchers studying climate-resilient crops”).  We cite Bain et al. (2019) as a reference for the claim that proponents use precision and non-transgenic status as arguments for why GE is less risky than GMOs. These authors explicitly trace how proponents utilize such claims. We also cite several other papers that more implicitly support this claim. |
|  | This paragraph frames the paper in terms of a dichotomy between rejection and acceptance which I think is rather unhelpful. The findings are much less dramatic and help us to better understand the connection between a number of variables which include the motivation behind the technology (to solve grand challenges and deliver public value or to serve corporate interests and deliver economic value), and the fit with values and ideas about the world people want to live in (industrialised agricultural systems). The paper’s contribution is valuable without having to sensationalise in terms of rejection/acceptance. More work needs to be done in this front end of the paper to ‘frame’ it in current policy realities about the failure of GE to solve the challenges it often claims to solve (i.e. feeding the world.) This paper might help frame the paper - Vanloqueren, G., & Baret, P. V. (2009). How agricultural research systems shape a technological regime that develops genetic engineering but locks out agroecological innovations. Research policy, 38(6), 971-983. This one would also help: Voegtlin, C., & Scherer, A. G. (2017). Responsible innovation and the innovation of responsibility: Governing sustainable development in a globalized world. Journal of business ethics, 143(2), 227-243. | We have modified the text throughout to ensure that our framing is not about whether public groups reject vs. accept GE, but rather, that our goal is to better understand the variables that might predict overall views on these technologies. We agree that this reframing better reflects the goal of our paper.  Thank you for this suggestion of the paper by Valoqueren and Baret (2009). We have also added this citation, as well as a related citation about technological trajectory lock-ins (Macnaghten 2018) in the previous section (1.1) to emphasize that technologies can depend on the broader societal context in which they emerge—and so too, we thus assert, public views can vary depending on understandings of what kind of agricultural systems appear these technologies appear more likely to end up supporting. |
|  | The claim at the start of paragraph 2 is not really accurate. There are lots of papers that do just this. One example the authors should draw on is the Royal Society (UK) public dialogue on GE which is highly relevant to this paper and has not been acknowledged - <https://royalsociety.org/topics-policy/publications/2018/genetic-technologies-public-dialogue/>.  1.1, second para – this is not accurate. GM technologies had the potential to deliver a much broader range of products than they did – again, Vanloqueren & Baret may help here. Para 3, less clear that what? I think this is one of the strengths of the paper: ‘minimal research has demonstrated the relationship between views on climate change and views on biotechnology’. This is a key feature and much more convincing than claiming the paper is about ‘rejecting or accepting’ GE. | Thank you for this suggestion. We have revised this paragraph to more accurately reflect the current state-of-the-art in research on public views on gene editing, and used this citation to strengthen our overarching argument—that while qualitative research has demonstrated that views on gene editing are connected to broader understandings of societal need and challenges—the quantitative perceptions work has not yet fully taken on these ideas. In fact, we now cite multiple findings from this study as justification of the need to quantitatively explore certain under-explored variables, such as questions of whether applications will support monocultures.  We have also clarified the point regarding what GM technologies had the potential to do, vs. what they actually did, emphasizing that they have largely been used for industrial agricultural production.  Lastly, we have also clarified the sentence “What is less clear…” so that it is easier to understand what comparison is being made. The sentence now reads: “While scientists studying these applications have proposed such climate-adaptive uses for gene editing, it is not clear whether such applications are persuasive or with whom.” |
|  | I’d like to see all the Hs listed to start with and then discussed in order before the sub-headings. How are the Hs connected? Iwould encourage the authors to use titles for these section headers rather than questions. | We tried to find a way to list the specific hypotheses (which we now call propositions, as we aren’t quite doing formal hypothesis testing) prior to explaining them. However we found that that approach was hard to understand, as the reader hasn’t yet been introduced to the concepts involved before reading each hypothesis. Our solution was to instead summarize our research questions before launching into a discussion of each specific hypothesis. We hope that our approach offers a balance of some initial framing and information, while also presenting each hypothesis at a point where the reader is equipped to understand it.  We have also changed the section headers here from questions to titles. |
|  | 1.1 title – this question doesn’t reflect the H. I’m not sure you’re looking at how people are swayed are you? | The language of ‘swayed’ as been modified to better reflect our goal (to understand the predictive power of different views and perspectives relating to views on gene editing). |
|  | The section numbering needs to be checked. Why 1.2.1 for H3? | This numbering has been corrected. |
|  | Section question for 1.2.1 is unclear – how does this relate to H3? You’re not really looking at this are you? The first para is particularly unclear. What is being traded off? It seems more about opting out of decisions or avoiding them rather than trading off. You state ‘We seek to explore the degree of such moral opposition to gene editing’ but this isn’t what you do. This section needs significant clarification. | We have modified the question at the beginning of this section (as noted above) so that it is a title rather than a question (“Exploring resistance to tradeoffs”).  We also have reworked the exposition in this section to clarify the relationship between ‘tradeoffs’, ‘opting out/resisting decisions’ and ‘moral opposition’. In summary, we clarify our aim of understanding the extent to which alleged ‘irrational absolutist objections’ to GE might indeed be connected to concerns about the broader food system in which the technology is embedded. |
|  | H2 and H3 – remove the term system critical and rephrase the Hs. H2 is rather confusing and consists of two sentences. It should be clear enough so a clarifying send sentence is not needed. | We have simplified this hypothesis to just one sentence, and removed mention of ‘system critical’, so that it now reads: “Participants with more negative views about corporations and the Green Revolution will have more negative views of gene editing.” |
|  | Methods - A lot depends on what information is provided to participants in terms of the green revolution. What was in the ‘tutorial’? More information needed here. How do you know their opting out isn’t just not fully understanding what the GR is? | In our survey, we first included a knowledge question in order to control for lack of familiarity with the Green Revolution: “I was familiar with the meaning of the term “the Green Revolution” before reading this question.” We found that results were similar for those who were familiar with the Green Revolution vs those who were not. We interpret this lack of difference to mean that participants who were unfamiliar with the Green Revolution may have utilized intuitive associations around the concepts of ‘fertilizers’ and ‘pesticides’ that we referenced in our tutorial, and reacted to those concepts even if they were not initially familiar with the Green Revolution. Based on the similarity of results across levels of familiarity, we included all responses in our results. |
|  | Table 1 – This needs further clarification. It’s not clear what you are telling us here and why it’s important. Trust is not discussed before this table. | Our aim was to build an explanatory model that might help us understand which variables might predict views on GE, and their relative explanatory power. To accomplish this, it was important to include—in addition to the novel predictors we sought to explore—other variables that have been shown to be significant via past studies. Thus, while trust isn’t the emphasis of our study, we included it to ensure robustness of the model. The ‘trust’ variable is discussed earlier in the methods section. To make sure the role of such a variable is easier to understand, we have reworked our description to emphasize this explanation. |
|  | Figures – I could not see these very well - the quality was quite poor. I’m sure this will be sorted out in the printing. | We will coordinate with journal to ensure that our figures are formatted properly. |
|  | The results and discussion are good. I would encourage the authors to push further in the discussion, taking the time and space to draw out the significance of the results for the literature. What do these results tell us about the theory and empirical reality? Without the theoretical framing at the start it’s a bit difficult at the moment, but I want to know why your results matter for the literature on responsible innovation, or public perceptions literature, for example. | We have expanded this section so that it better links with the new up-front theoretical framing, and draws out these linkages more fully. We explain that these results really shed light on the importance of considering that views on GE are not only shaped by thinks like understandings of the technology, specific concerns about environmental and health risks, etc., but rather, are linked to broader questions about the visions that people have about what food systems might look like in the future.  We also argue that our efforts to incorporate such value-based considerations offer one way to make risk perceptions research more informed by responsible research and innovation. Building off of this, we propose a couple methodological approaches for the risk perceptions field to better incorporate RRI principles. |
|  | The policy implications section is really good and I would recommend the authors expand this section considerably – this is one of the most important sections where you reflect on what your research means. However, the Norwegian Biotech Advisory Board reference is taken rather critically given the criticisms that have started to emerge. See -Kjeldaas, S., Antonsen, T., Hartley, S., & Myhr, A. I. (2021). Public Consultation on Proposed Revisions to Norway’s Gene Technology Act: An Analysis of the Consultation Framing, Stakeholder Concerns and the Integration of Non-Safety Considerations. Sustainability, 13(14), 7643. | Thank you for this feedback. We have noted the useful paper you cited that pointed out some criticisms of the NBAB approach. We also have added some additional reflections on how our findings might be useful for informing proponents’ activities. |
